# Time and Repetitions Needed to Train Patients with Knee Pain on a Home Exercise Program: Are Learning Styles Important?

**DOI:** 10.7759/cureus.6988

**Published:** 2020-02-14

**Authors:** Serap Satış, Celal Erdem

**Affiliations:** 1 Physical Medicine and Rehabilitation, Harran University Faculty of Medicine, Şanlıurfa, TUR; 2 Physical Medicine and Rehabilitation, Harran University Hospital, Şanlıurfa, TUR

**Keywords:** home exercise programme, knee pain, quadriceps isometric exercises, heel slide exercises, adductor isometric exercises

## Abstract

Objective

This study was designed to identify the amount of time and number of repetitions needed to explain a home exercise program recommended for most of our patients, as well as to gauge how many items patients managed to remember at their 15-day follow-up. We also considered whether the learning method had any effect on these results.

Methods

Sixty-two patients with mechanical knee pain who were admitted to our clinic were included in this study. Patients were categorized into the following three groups: group 1 with a dominant physical learning style, group 2 with a dominant auditory learning style, and group 3 with a dominant visual learning style. Heel slide, quadriceps isometric, quadriceps stretching, adductor isometric, abductor isometric, and quadriceps isotonic exercises were explained and demonstrated to all patients by the same physiotherapist, and the required time (in seconds) and repeats of exercises until the patients learned them were recorded. Remembered/forgotten exercises at the follow-up, which occurred 15 days later, were identified.

Results

A statistically significant difference was observed between groups in terms of how many seconds were needed for learning the quadriceps isometric exercises (p: 0.042). In the inter-group comparison, the difference was significant when groups 2 and 3 were compared (p: 0.046). There was a significant difference between groups in terms of how many repeats were needed for learning heel sliding (p: 0.000). Moreover, there was a significant difference between group 3 and groups 1 and 2 in the inter-group comparison (p: 0.000, p: 0.000). There was also a significant difference between groups in terms of recalling the adductor isometric exercises. Patients in group 2 were able to fully recall all these exercises.

Conclusion

It was found that the quadriceps isometric, heel slide, and adductor isometric exercises were more quickly learned, while the quadriceps stretching exercise was forgotten. We concluded that learning style is not highly important in exercise learning or recall.

## Introduction

Therapeutic exercise can be defined as body movements performed for correcting any disorder, improving locomotor system performance, or sustaining an optimal condition. The movements are carefully selected special exercises for various parts of the body or certain muscle groups. These include exercises to improve joint range of motion, stretching, strengthening exercises, aerobics, and balance-coordination exercises. A home exercise program consists of selected disease-specific exercises performed at home. These can be taught by a physiotherapist or doctor and presented as written brochures or video images.

Knee pain is among the leading causes of hospital admissions, as the knee is the biggest synovial joint and has a complex structure. Knee pain may arise due to mechanical, inflammatory, or infectious agents. The leading cause of mechanical knee pain is osteoarthritis, and exercise is important in the treatment of knee osteoarthritis [1,2]. Other causes of mechanical knee pain are patellofemoral syndrome, infrapatellar bursitis, synovial plica, patellar tendinitis, quadriceps tendinitis, Osgood-Schlatter syndrome, osteochondritis dissecans, meniscus, and connective tissue disorders. Fear of using the knee due to pain causes muscle weakness; this results in disability because the knee is one of the joints carrying the bodyweight [3,4]. Patients with knee pain must perform special exercises for increasing muscle power, the joint movement range, and joint stability. Many studies have examined patient compliance with home exercises; however, no data have been published regarding which exercises are easily learned and remembered by patients [5,6]. Exercises that can be easily learned and remembered are generally easier to perform.

The objective of this study was to identify how much time (in seconds) and how many repetitions were needed by the patients for learning a home exercise program and how many items relating to the program are remembered by them at a 15-day follow up. Another objective was to understand whether learning style had any effect on those results.

## Materials and methods

A total of 62 patients who were admitted to the Physical Medicine And Rehabilitation Clinic at the Harran University Faculty of Medicine, Şanlıurfa, Turkey due to knee pain and mechanical knee pain were included in the study. Patients with inflammatory/infectious pain and patients who had undergone knee operation were excluded. Patients were informed about the study, and written consents were obtained. This study was approved by the Ethics Committee of the Harran University Faculty of Medicine.

Patients were divided into three groups according to their dominant learning style: group 1 had a dominant physical learning style, group 2 had a dominant auditory learning style, and group 3 had a dominant visual learning style. The same physiotherapist explained and demonstrated the heel sliding, quadriceps isometric, quadriceps stretching, adductor isometric, abductor isometric, and quadriceps isotonic exercises; the time in seconds and number of repeats needed for learning these exercises by the patients were recorded. Exercises that were remembered/not remembered by the patients at the follow-up after 15 days were identified. Exercises involving three sets of 10 repeats for five days every week were recommended to patients.

The BIG 16 Learning Style Inventory, developed by Şimşek, was used for identifying the learning styles of included patients [7]. This inventory measures three learning styles: physical, auditory, and visual. It is composed of a total of 48 items, with 16 items for every style. A 5-point Likert scale was used for the evaluation of each item, with options of “Strongly agree” (2), “Agree” (1), “Neither agree nor disagree” (0), “Disagree” (−1) and “Strongly disagree” (−2). In this inventory, the mathematical sum of every number for each style is written in the space under the relevant category. If the item scores are between 7 and −7, the respondent is not considered to have the associated style; if they are in the range of 8-32, it means that the participant has that style. The exercises recommended as the home exercise program are described in Table 1. In this study, associations between groups in terms of how many seconds and repeats were needed to teach each exercise and the ability to recall the exercise after 15 days were examined.

**Table 1:** Exercises recommended as part of home exercise program

Exercise	Description
Heel sliding exercise	Lying with outstretched legs on a flat surface, the knee is pulled to the thorax and moved through hips without lifting heel from the floor. The other leg must be in extension
Quadriceps isometric exercise	Lying with outstretched legs on a flat surface, the knee is pressed on the floor. Muscle contraction must be felt
Quadriceps stretching exercise	Lying prone on the floor, a band on the ankle of the exercised leg is stretched until knee flexion
Adductor isometric exercise	Lying with outstretched legs on a flat surface, two knees are pressed on each other. Must pause for five seconds. Muscle contraction must be felt
Abductor isometric exercise	Lying with outstretched legs on a flat surface, two knees are contracted and moved away from each other. Must pause for five seconds. Muscle contraction must be felt
Quadriceps isotonic exercise	Sitting up straight and swinging legs, a leg is elevated from knee and knee is straightened. Must pause for five seconds in this position and the leg is slowly descended

## Results

Group 1 was composed of 15 patients (7 female, 8 male) with a mean age of 40.93 ±19.91 years; group 2 comprised 31 patients (19 female, 12 male) with a mean age of 42.96 ±15.67 years; and group 3 included 16 patients (8 female, 8 male) with a mean age of 40.56 ±14.46 years. Table 2 lists the time (in seconds) needed for learning exercises according to the participants’ dominant learning style, while Table 3 lists the number of repetitions needed for learning. Table 4 lists the remembered/not remembered exercises 15 days later at the follow-up.

**Table 2:** Time needed for learning the exercises SD: standard deviation

	Group 1, mean ±SD	Group 2, mean ±SD	Group 3, mean ±SD	P-value
Quadriceps isometric exercise, seconds	2.00 ±1.41	2.75 ±1.18	2.41 ±1.23	0.264
Quadriceps stretching exercise, seconds	2.46 ±1.30	3.18 ±1.55	2.38 ±1.14	0.128
Heel sliding exercise, seconds	1.13 ±0.35	2.68 ±1.88	1.12 ±0.34	0.000
Adductor isometric exercise, seconds	1.53 ±0.63	2.00 ±0.00	1.93 ±1.03	0.203
Abductor isometric exercise, seconds	1.53 ±0.63	2.18 ±0.10	1.96 ±0.94	0.063
Quadriceps isotonic exercise, seconds	1.00 ±0.00	1.18 ±0.40	1.12 ±0.34	0.246

 

**Table 3:** Number of repetitions needed for learning the exercises SD: standard deviation

	Group 1, mean ±SD	Group 2, mean ±SD	Group 3, mean ±SD	P-value
Quadriceps isometric exercise	25.00 ±14.39	29.3 ±79.28	26.90 ±17.57	0.721
Quadriceps stretching exercise	28.66 ±14.32	32.81 ±11.96	30.16 ±14.24	0.716
Heel sliding exercise	12.00 ±4.14	15.62 ±8.13	11.93 ±4.59	0.089
Adductor isometric exercise	19.33 ±13.34	17.50 ±6.83	22.90 ±11.60	0.256
Abductor isometric exercise	19.33 ±13.34	19.37 ±5.73	21.61 ±10.67	0.696
Quadriceps isotonic exercise	11.33 ±3.51	13.75 ±2.23	11.12 ±3.381	0.042

 

**Table 4:** Number of patients who remembered/not remembered exercises at 15-day follow-up

		Group 1, n	Group 2, n	Group 3, n	P-value
Quadriceps isometric exercise	Did not remember	1	0	4	2.298
	Remembered	14	16	27	
Quadriceps stretching exercise	Did not remember	7	14	19	0.052
	Remembered	8	2	12	
Heel sliding exercise	Did not remember	0	0	2	0.356
	Remembered	15	16	29	
Adductor isometric exercise	Did not remember	7	0	5	0.004
	Remembered	8	16	26	
Abductor isometric exercise	Did not remember	6	6	11	0.956
	Remembered	9	10	20	
Quadriceps isotonic exercise	Did not remember	0	0	0	

There was a significant difference between the groups in terms of how many seconds were needed for learning the quadriceps isometric exercise (p: 0.042). Moreover, there was a significant difference between groups 2 and 3 in the inter-group comparison (p: 0.046). There was also a significant difference between groups in terms of how many repeats were needed for learning the heel slide exercise (p: 0.000); a significant difference was observed between group 3 and groups 1 and 2 in the inter-group comparison (p: 0.000, p: 0.000).

There was a significant difference between the groups in terms of remembering the adductor isometric exercise. All patients in group 2 remembered this exercise. It was observed that all patients remembered the quadriceps isometric exercise; however, they had difficulty recalling the quadriceps stretching exercise. The heel sliding exercise was remembered by all patients in groups 1 and 2, whereas two patients in group 3 did not remember this exercise. It was concluded that the patients in group 2 were better than those in the other groups in terms of recalling the exercises.

Table 5 lists the education status of the group members. There was no significant difference between the groups in terms of education status (p: 0.080)

**Table 5:** Education status of the group members

	Group 1, n	Group 2, n	Group 3, n
Illiterate	1	7	7
Primary school graduate	4	1	8
Middle school graduate	4	2	6
High school graduate	1	1	7
University graduate	5	5	3

## Discussion

The knee is the biggest synovial joint in the human body, and it carries the body weight. Due to its complex structure, many factors may cause knee pain. Knee pain is among the locomotor system disorders, which are the leading causes of admissions to hospitals. It has been observed that knee pain is the leading cause of loss/disruption of employment [8].

Atrophy and weakness due to lack of use are often observed in the quadriceps muscle, which is one of the most important static stabilizers [9,10]. This also causes pain chronization such as joint pain, stiffness, and decreased joint mobility. Isometric, isotonic, and isokinetic exercises for increasing muscle power must be recommended along with medical treatment in cases of knee pain. These exercises are usually suggested as part of home exercise programs in clinics. Exercises can be taught by a physiotherapist or doctor; they can also be recommended with visual resources such as written brochures or video images. It has been shown that home exercise programs are effective for controlling pain, increasing muscle power, and improving joint movement range and functional capacity [11].

Patients’ consciousness, learning skills, literacy, social status, and willingness to exercise affect their home exercise performance. The most effective program will consist of safe, individualized exercises in accordance with the patient’s objectives. Safe exercise must be prescribed after considering the patient’s perceptions and fears about exercise. Motivation increases the will to learn and perform exercises. The problem the exercise is intended to address must be explained to the patient; the aim of the exercises must be stated, and their benefits must be highlighted. Patients must be informed in advance about the exercises, and the trainer must reinforce the training by showing the patients how to perform them on their bodies if necessary. Visual brochures or video records prepared for home exercise programs may be provided. According to clinical experience, patients do not often use brochures. Indicating the performance frequency and repetition count is another especially important point. The patients must be called for follow-ups, and the exercise efficiency must be evaluated; patients should be reminded of elements they have forgotten, and they must be corrected when exercises are performed in the wrong way. In light of the findings of this study, we think that prescribing easily learned and remembered exercises to patients is more effective than including exercises that may be harder to learn and recall. The quadriceps isometric exercise is a case in point; it was learned more quickly and with fewer repeats than the other exercises and completely remembered by all the patients, and it would top our list of exercise recommendations. Quadriceps stretching exercise must be performed only under supervision at the hospital, as this exercise seems harder to remember.

It has been stated that exercise prescriptions must be properly individualized for patients with chronic locomotor system disorders [5,6]. Evidence levels of results in studies investigating the effects of exercise on pain control are low [12]. However, it has been found to be effective in pain control in some studies [12-14]. It seems that its effect on pain is significant, and this is not related to frequency and duration; exercise is even effective when it is performed once per week [12]. Studies related to the exercise session duration and frequency are not widely available in published literature. There is no consensus on this topic as the results of the conducted studies have been controversial [15].

Pain and morning stiffness decreases in 66.7% of patients performing a home exercise program. It has been reported that the reasons for not performing exercises are pain, lack of belief in the exercise, lack of free time, and inadequate information [16-19]. However, there has been no study as to which exercises are easily learned or easily recalled. Our study aimed to investigate this issue, and it was concluded that the adductor isometric, quadriceps isometric, and heel sliding exercises were easily remembered by the patients. It was observed that the quadriceps stretching exercise was the most difficult to remember. This may be due to the negative effect of chronic pain on the cerebellum and the resulting neuromuscular coordination.

The home exercise program we analyzed is provided free of charge. It is as effective as electrotherapy when regularly performed [20]. However, many patients consider exercise to be ineffective and tend to quit the program. Most of the previously performed studies have investigated patient compliance with home exercise programs, and they have usually highlighted the importance of the patient-physiotherapist relationship. It has been concluded that patient compliance with exercise decreases without physiotherapist supervision [17]. One study suggested that visual brochures are enough to motivate patients and video or voice records are not needed [16]. We did not recommend any materials like videos, audio recordings, or brochures.

In some studies, it has been observed that a higher level of education increases compliance with home exercise programs [18,19]. However, some studies have found reduced compliance with more education [21]. We investigated the effect of learning style on a home exercise program and the extent of compliance. We concluded that learning style is not highly important for exercise learning or recall. Fatigue and complexity of exercises have been reported as factors decreasing patient compliance. In light of the results of our study, we believe that prescribing simple exercises to patients may improve compliance.

The main limitation of this study was the small sample size. Studies involving larger sample sizes are needed to further explore the effectiveness of exercise programs in the rehabilitation of patients with knee pain.

## Conclusions

In specific cases, exercise must be the first measure taken to treat knee pain. However, it is frequently neglected. Although compliance with exercise programs is found to good in the initial phases, it decreases gradually over time. Increased pain due to improperly performed exercises, patient perceptions, prescribing exercise without considering patients’ social status, and patients forgetting exercises are the causes of compliance reduction.

In this study, we concluded that the quadriceps isometric, heel sliding, and adductor isometric exercises, which can be learned more swiftly and are easy to remember, should be more frequently suggested. Performing quadriceps stretching exercises under supervision is more appropriate because they are more difficult to remember. We also deduced that learning style is not highly important for exercise learning and recall.
